# Multimorbidity care during TB treatment: no longer if, but how

**DOI:** 10.5588/ijtldopen.26.0337

**Published:** 2026-09-15

**Authors:** C. Calderwood, E. Marambire, O. Mufare, S. Bernays, K. Kranzer

**Affiliations:** 1 National Heart and Lung Institute, Imperial College London, London, UK;; 2 The Health Research Unit Zimbabwe, Biomedical Research and Training Institute, Harare, Zimbabwe;; 3 Clinical Research Department, London School of Hygiene & Tropical Medicine, London, UK;; 4 University of Sydney, Sydney, Australia;; 5 Division of Infectious Diseases and Tropical Medicine, University Hospital, LMU Munich, Munich, Germany.

**Keywords:** tuberculosis, integrated care, service delivery, TB survivors, chronic respiratory disease

Dear Editor,

The recent work by The Kenya, Uganda, Zambia and Zimbabwe TB Disability Study Group, together with the accompanying Editorial, frames an important agenda for NTPs, funders, and the wider TB community on implementation of integrated, multimorbidity-centred TB care.^1–3^ In 2024, more than 10 million people developed TB, with incidence concentrated among men, people of working age, and socioeconomically deprived communities.^1,4^ TB seldom occurs alone, but does so within a context of chronic disease and vulnerability.^5^ Established non-communicable diseases (including diabetes, chronic lung disease and mental health disorders); infections including HIV; undernutrition; and clustered risk factors (including alcohol, tobacco and substance dependence) increase TB risk and complicate treatment.^6^ These often coexist as TB multimorbidity (≥2 long term conditions in a person with TB^6^) and the relationship is bidirectional – risk factors and comorbidities drive TB and TB further entrenches and amplifies comorbidities and their drivers and results in additional morbidity.

In their recent work Banda et al. confirmed that these syndemic interactions and consequences of TB manifest as a high prevalence of end-of-treatment multimorbidity, and identified that comorbidities are not routinely detected or treated during TB treatment.^2^ They then showed that comorbidity assessment is feasible within four African National Tuberculosis Programmes (NTPs).^1^ This is proof-of-concept that multimorbidity-oriented care is needed and achievable in high-burden, resource-limited settings. The authors concluded that integrated screening and referral for multiple comorbidities among people with TB resulted in major reductions in symptoms and disability and improved return to work. These are, however, expected outcomes of effective TB treatment. The conclusions are based on baseline comparisons, which cannot distinguish screening and referral effects from usual TB treatment trajectories, and comparison to an earlier cohort which is limited by confounding and temporal trends. The authors also place substantial weight on symptomatic improvement which, whilst important, is subjective.^7^ In determining the impact of TB-comorbidity care objectively measured outcomes are important; for long-term, usually irreversible, conditions (such as diabetes, HIV and hypertension), these should focus on disease control rather than prevalence. Nonetheless, expert consensus has established that people with TB should be routinely screened and offered care for comorbidities, including these conditions, during TB treatment, and the package components are well evidenced.^8^ Smoking-cessation interventions are feasible and effective.^6^ Nutritional supplementation reduces mortality.^9^ Diabetes care improves glycaemic control and outcomes.^6^ Interventions for common mental health disorders improve adherence, outcomes and symptoms.^6^ The case for early, integrated assessment and care should not be weakened by acknowledging that this study design cannot quantify the benefit of the model above TB treatment alone.

The individual-level clinical effectiveness outcomes assessed by Banda et al. exclude other potential impacts of integrated TB care (see Figure). First, addressing comorbidities and risk factors may improve WHO-defined TB treatment outcomes.^10^ Second, current service configurations often fail to meet the needs and priorities of people with TB; whereas responsive, person-centred care that improves client-provider relationships can build trust and engagement during and beyond treatment.^11^ Third, reorienting care from a vertical programme for a single, stigmatised infection towards integrated, primary health care may defuse blame and stigma from providers and within communities. This may have multiple benefits including earlier and sustained care engagement by people at risk of and with TB, disrupting pathways to infection and poor outcomes.^5^ Integrated TB services may be cost-effective through both improved health outcomes and economy-of-scope. Further, integrated care may serve as an investment in prevention with positive sequalae beyond the current evidence timeframe – including aversion of catastrophic costs. Deliberately expanding integrated care beyond treatment of disease to holistic healthcare underscores TB’s curability and invests in TB survivors’ restored productivity and flourishing health, reframing social and relational narratives of TB. These plausible impact mechanisms remain largely unmeasured and should be considered in future, including longer-term and indirect sequalae.

**Figure.**
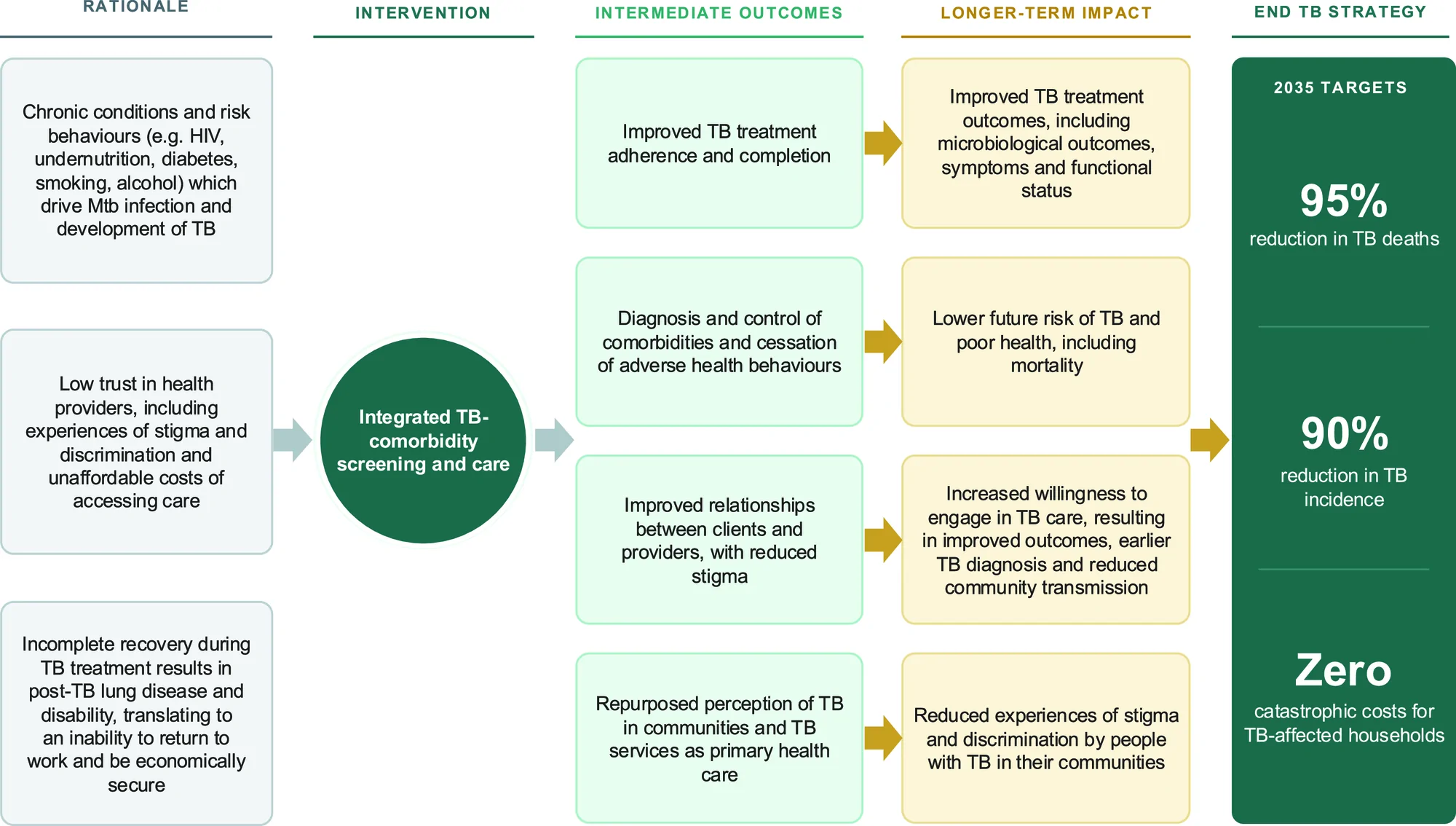
Rationale and potential impacts of multimorbidity-centred TB care. Improved functional status (enabling return to work) and reduced stigma and discrimination (supporting access to employment) are hypothesised to reduce catastrophic costs for TB-affected households. Arrows denote hypothesised pathways. Mtb = Mycobacterium tuberculosis

An important finding of this study was the variable rate of referral completion, which speaks to optimal care configuration: within-facility referrals achieved near-complete linkage, but external referrals were often incomplete.^1^ This is consistent with substantial evidence that one-stop-shop services outperform separate referral pathways. Even greater gains than those reported by Banda et al. may be possible through the development of truly integrated, one-stop-stop models of TB multimorbidity care.^12^ Further evidence is also needed on implementation outcomes, including training and delivery requirements, acceptability, adoption and scalability.

Banda et al. should be commended for demonstrating that operationalising multimorbidity care within TB services is possible, and for foregrounding the role of NTPs in this agenda. The evidence on the individual components is clear: the question is no longer whether to address multimorbidity during tuberculosis treatment, but how to integrate and deliver services to achieve better health, wellbeing and economic security for people with TB. We are testing this through a hybrid type 2 cluster-randomised trial of integrated multimorbidity services (addressing nutrition, diabetes, HIV, smoking, alcohol, mental health and functional impairment) delivered within routine TB services in Zimbabwe.

Funding: this work was supported by the Medical Research Council (UKRI2113: Rehabilitation with embedded mental health and diabetes services to address multiple long-term conditions among people with tuberculosis [RESTORE]) as part of the Global Alliance for Chronic Diseases Multiple Long Term Conditions Research Programme.
